# The impact of anti-COVID measures on accommodation performance

**DOI:** 10.12688/openreseurope.16566.2

**Published:** 2024-07-29

**Authors:** Milada Šťastná, Kateřina Ryglová, Antonín Vaishar, Andrea Králíková

**Affiliations:** 1 Department of Applied and Landscape Ecology, Mendel University in Brno, Brno, South Moravian Region, 61300, Czech Republic; 2 Department of Marketing and Trade, Mendel University in Brno, Brno, South Moravian Region, 61300, Czech Republic

**Keywords:** tourism, COVID-19, regional differences, mass accommodation facilities, Czechia

## Abstract

**Background:**

This article analyses mass accommodation facilities to examine the development of Czech tourism during the COVID-19 pandemic of 2020 and 2021.

**Methods:**

The questionnaire survey was carried out in March 2021 in mass accommodation facilities. 131 responses were received from hotels and guesthouses throughout Czechia, which represents a return rate of 20%. Data were processed using Pearson's chi-square test to determine the effect of changes in facility type and category, quality, location, and primary focus before the outbreak of the pandemic. A statistical analysis of data on overnight stays was also used with data from the public database of the Czech Statistical Office.

**Results:**

The study confirmed the increasing importance of domestic tourism and the greater sensitivity of destinations dependent on foreign tourists. Future strategies should be associated with destination rather than corporate management. The challenges are linked to the strengthening of environmentally friendly and sustainable tourism. To what extent the post-covid situation will return to the original model and to what extent it will at least partially reflect the changes that took place during the crisis period is a question for future research.

**Conclusions:**

Moving from operational to strategic measures would be advisable.

## Introduction

Since the beginning of the 21st century, globalization, the opening of borders, the growth of the middle class in China, India, and other developing countries, and the proliferation of air transport have encouraged the rapid development of international tourism ( Lukianenko *et al.,* 2019). However, the COVID-19 pandemic in the early 2020s severely affected this development. As measures to control the pandemic, borders were periodically closed, most airlines came to a halt and international tourism almost stopped; this dealt a severe blow to the global tourism industry.

However, not all tourism segments were equally affected. In our preliminary findings ( Vaishar & Šťastná, 2022), we found that tourism suffered less of a decline in rural micro-regions, which are less dependent on international tourism than major cities, UNESCO World Heritage Sites, conference centres, and great spa towns.

National governments, including the Czech government, took several measures to save the tourism industry, particularly to preserve jobs and businesses ( Assaf & Scuderi, 2020). Regional governments concentrated on supporting destination management ( Pillmayer *et al.*, 2021), and individual entrepreneurs sought new business opportunities, often using online technologies.

Our article documents the number of visitors to tourist destinations in Czechia to confirm or refute the hypothesis that rural areas less dependent on foreign tourists were less affected by the disruption in tourism caused by the pandemic. We also endeavor to predict whether international tourism will return to its pre-pandemic level or if domestic rural tourism trends are long- or medium-term. Our paper uses a questionnaire survey. The survey's primary goal was to illustrate the changes in hotel strategies, including pricing and sales approaches, hotel segments, and adjustments in product portfolios. Additionally, we analyzed the impact on the company’s operations, particularly focusing on the number of employees and costs compared to the pre-pandemic period. Moreover, we investigated safety requirements.

### Theory

In a post-productive (consumer) society, tourism is a rapidly developing sector of the national economy ( Comerio & Strozzi, 2019), promoting economic growth ( Li *et al.*, 2018). In addition to owning tourism facilities, several other service and supply sectors are also linked to tourism ( Pascariu & Ibănescu, 2018). However, infrastructure built primarily for tourists can also benefit local people. Tourism contributes to a country’s economy, and this contribution is significant in some countries. In southern European countries, tourism accounts for between a quarter and a fifth of gross domestic product (GDP). The share of tourism in the Czech economy is lower and has been declining recently. Nevertheless, it still accounts for almost 8% of the GDP ( Portal Finlord, 2020). Although Czechia is not dependent on tourism, the sector is still important.

However, as smaller national businesses and global chains play important roles in the sector, tourism plays an important role in the local economy ( Suhel & Bashir, 2018). To some extent, employment in tourism replaces job losses in manufacturing, especially in peripheral rural areas ( Santos *et al.*, 2019). However, it should be noted that tourism does not require highly skilled workers, is characterized by low wages, and seasonality, and has a demanding workload. As a result, it cannot improve a locality’s educational or social situation, and its benefits lay more in its multiplier effect and cultural role.

The classical analysis of tourism deals with the three factors that influence tourism. These factors are localization (natural attractions, historical and cultural heritage), infrastructural (capacity and level of accommodation and catering facilities, transport, and other infrastructure), and implementation (institutional and organizational conditions for tourism development and the human factor). Also, various categories of tourists (demographic, social, or hobby), seasonality, and other aspects are considered. It is important to distinguish between domestic and foreign tourists.

Tourism is developing as an economic factor under the ideological influence of a neoliberal economy focused on continuous growth ( Higgins-Desbiolles, 2020). Like other industries, tourism encounters territorial and social barriers. Overtourism is a problem in particularly exposed destinations, which are predominantly cities but also some rural resorts ( Dodds & Butler, 2019). The research question relates to the problem of sustainable tourism ( Hall, 2019).

However, tourism is not just a sector of the economy. It is also an activity that expands people’s knowledge of foreign regions. This can result in greater tolerance between people of different cultures, the personal development of tourists and locals, and the consolidation of local, regional, national or European identities ( Gertner, 2019). Similar to the jobs secondarily created by tourism, the benefits of these intangible aspects may be more significant than those of tourism itself. In this sense, tourism plays a significant role in the community life of destinations ( Yu *et al.*, 2018).

Cultural tourism fulfils this role in particular ( Richards, 2013). United Nations World Tourist Organization defines cultural tourism as follows: Cultural tourism is a type of tourism activity in which the visitor's essential motivation is to learn, discover, experience and consume the tangible and intangible cultural attractions/products in a tourism destination. It follows that cultural tourism is mainly connected with cognitive function. It is about getting to know the natural and cultural heritage, the local population, their customs and culture. These are often recognized through events and/or gastronomy. An important trend is creative tourism, in which tourists plan and organize their own trips. This allows you to penetrate more into local cultures than tours organized by travel agencies. An important trend is creative tourism, in which tourists plan and organize their own trips. This allows you to penetrate more into local cultures than tours organized by travel agencies. Of course, the creativity of tourists must be reflected in the creation of creative destinations ( Richards, 2020).

Unfortunately, tourism is also a very risky sector ( Alvarez *et al.*, 2022). It is sensitive to changes in natural conditions and natural disasters. It is also subject to social risks such as economic downturn, reduced security, and, in extreme cases, war. In addition, it is subject to fashion. The COVID-19 pandemic was an extreme example of such a development due to its global effects ( Abbas *et al.,* 2021). The pandemic resulted in a reduction in overall tourism and redirection of tourists.

The COVID-19 pandemic primarily and secondarily affected tourism in 2020 and 2021. We can consider tourists’ fears of the disease as the primary influence. Tourism was a factor in the pandemic, as its rapid spread worldwide was caused by tourism ( Hoarau, 2022). The consequences of anti-pandemic measures are a secondary impact. There were various restrictions, such as restricting or closing borders, restricting air traffic, and hygiene measures in destinations ( Bruinen de Bruin *et al.,* 2020). Another important issue was the acceptance of these measures by tourists, providers, and locals ( Dohle *et al.*, 2020). In addition, some tourism facilities temporarily or permanently ceased operations, lay off staff, and cannot receive visitors. A comparison of the consequences of the crisis in 31 European countries was dealt with by Roman *et al.* (2022). Czechia was included together with Slovakia, Croatia, and Slovenia in the cluster of countries with the greatest restrictions on air transport. However, due to the location of the country in the middle of Europe and the relatively low contribution of tourism to the creation of GDP, Czechia does not belong to the most affected countries in a European comparison.

The tourism industry had to adapt to the current situation ( Sanabria-Díaz *et al.*, 2021). New products were developed, new target groups were contacted and new digital methods were employed. New, more adaptable companies can build on those that are now defunct. As with any crisis, the COVID-19 pandemic has cleaned the market; however, some congested are happy that tourism has slowed down ( Amrhein *et al.* (2022). Ntounis *et al.* (2022) examined tourism’s resilience to pandemics and constructed a novel Business Resilience Composite Score.

The differences between urban and rural tourism were important for our study. Above all, urban tourism usually has a greater concentration of attractions and better infrastructure; and is often subject to global trends. Rural tourism is more linked to the region and locality, local customs, food, landscape, and open-air activities ( Rosalina *et al.*, 2021); it can support both local and regional identities ( Väisänen & Törn-Lapio, 2018).

## Methods

### Ethical statement

This study received ethical approval from the Ethics Committee of Mendel University, Brno under ethical approval number (No 13). Written informed consent was obtained from all participants by completing the survey distributed.

### Study design and setting

Operating according to COVID-19 restrictions required unprecedented versatility from tourism service providers. This paper examines the impact of the COVID-19 pandemic on the hotel industry, and the findings could help develop new strategies for hotels. Czechia is a developed country located in the centre of Europe, with an advanced economy based on manufacturing and services; it was ranked 9 ^th^ by the Global Peace Index in 2021 ( Global Piece Index, 2021). The most important tourist destinations in terms of the number of overnight stays in 2021 were: Prague (5,257,000), Karlovy Vary Spa (2,444,000), Giant Mts. (2,187,000), High Ash Mts. (1,319,000), Palava and Lednice-Valtice (1,072,000), Brno and surroundings (947,000), and Zlín and Luhačovice (822,000).

The South Moravian region includes five tourism areas covered by destination management: Brno and surroundings, Moravian Karst, Moravian Slovakia, Znojmo with Thaya valley and Pálava Highlands with the Lednice-Valtice area. Apart from Brno, other areas can be considered rural. The region must be able to offer a wide spectrum of tourism combinations. The city of Brno, with five public and one state university, is an important center for conference tourism and a major cultural hub with the functionalist Villa Tugendhat, which is part of the UNESCO World Heritage Site. It also has a wide range of accommodation and catering options with a capacity of 12,770 beds.

The natural attractions of the region are represented by the Thaya Valley National Park, the protected landscape areas Moravian Karst, White Carpathians and Pavlovské vrchy hills, as well as the Lower Morava Biosphere Reserve. Water recreation is possible at the Nové Mlýny, Brno, Vranov and several smaller reservoirs. An important attraction is the Austerlitz battlefield east of Brno. Part of the UNESCO World Heritage Site is the composite landscape of the Lednice-Valtice area. The region lacks mountain resorts, but winter recreation takes place in some places, including the lowest area of Alpine skiing in Europe in Němčičky (180 m a.s.l.). The surroundings of Brno, southwestern Moravia and other parts of the region are areas for second housing. Tourism is supported by one of the most perfect hiking trails in the world. In 2019, there were 43,000 km of marked hiking trails in Czechia and another thousands of kilometers of bike trails, ski trails, riding trails and educational trails.

A special comparative advantage of the region compared to other regions of the Czech Republic is its wine culture, linked to surviving folklore. 96% of the country's vineyards are located here. Wine is connected with a number of other activities - team building events, cycling tours, ethnographic events. In its modern form, it includes the entire cycle from growing vines, through wine production, its distribution, consumption, guest accommodation and accompanying events. Wine culture is linked to surviving folklore, especially dulcimer music, dances, ethnographic events, folk literature, costumes and customs. Intangible UNESCO World Heritage items are also linked to folklore, namely the male dance Verbuňk, the ethnographic festival Ride of the Kings and the folk craft of blueprint.

The architectural heritage is represented by a wide range of castles (Špilberk, Veveří, Pernštejn, Bítov and many others), chateaux (e.g., Lednice, Valtice, Vranov. Mikulov, Milotice, Kunštát), historic city centers, monasteries (Předklášteří, Dolní Kounice, Znojmo, Rajhrad), churches, places of pilgrimage (Křtiny, Blatnice), and technical monuments (Old smelter in Adamov, Baťa channel, windmill Kuželov). There are also many Jewish monuments in the region, especially synagogues and cemeteries (Boskovice, Mikulov, Dolní Kounice, Ivančice). Spas (Lednice, Hodonín), wellness facilities (Pasohlávky, Kuřim, Vyškov), and golf resorts (Kaskáda Kuřim, Austerlitz Golf Resort Slavkov) have been growing recently. In the forests west of Brno, there is the Masaryk Circuit motoring area.

The South Moravian countryside is therefore a potentially attractive place for the development of tourism. However, there are several significant barriers. One of them is significant seasonality. Another barrier is the still lagging infrastructure compared to Western Europe. The road network is very dense, but often in poor condition. Parking lots and especially their amenities are often missing. The frequency of public transport is sufficient: every populated place in the region must be served by public transport at least six times a day on weekdays, and three times a day on holidays and weekends. However, the culture and speed of public transport travel cannot compete with individual transport. Information services and especially the cooperation of providers in individual destinations could also be improved.

The numbers of overnight stays divided into resident (domestic) tourists and non-residents were taken from the public database of the Czech Statistical Office. This data only captures tourists who spent at least one night in a collective accommodation facility. They therefore do not include tourists in individual facilities such as private apartments or cottages, which are, however, probably used mainly by domestic tourists.

### Participant selection and data tools

A questionnaire survey was conducted in lodging establishments to understand the implications of the pandemic for the tourism industry and the strategy providers adopted. The survey included questions related to changes in consumer behavior from the lodging industry's perspective, changes in occupancy, cost reductions, and number of employees. It also covered changes in sales and pricing strategies and whether establishments were targeting the same clientele as before the pandemic or whether their segment had changed.

### Data collection

Data regarding the number of overnight stays in Czechia as well as South Moravia from 2012 to 2021 were accessed via the Public Database of the Czech Statistical Office ( https://vdb.czso.cz/vdbvo2/faces/en/index.jsf?page=statistiky#katalog=31743). In the section Occupancy in Collective Accommodation Establishments there is an option of displaying data on national, regional and local level.

Primary data were collected in March 2021 via a direct mail survey. The questionnaire (in Czech as well as translated into English is available;50) was sent to 666 lodging establishments in Czechia according to their Hotelstars classification (Association of Hotels and Restaurants of the Czech Republic; hsukatalog.cz, 2021 ^1^). We used quota sampling based on the official accommodation category from the Hotelstars (2021). In total, we received responses (via email) from 131 hotels (54.2%) and guesthouses (45.8%) across Czechia, ranging from one to five stars. This was a return rate of approximately 20% (see Table 1). Since the questionnaire did not contain any questions that indicated a specific lodging establishment, the data were completely anonymous. An Excel spreadsheet was used to visualize the data ( Šťastná *et al.*, 2023). From this visualization, we could see that no data was missing.

**Table 1.** Structure of the respondents (accommodation providers/lodging establishments).

	Number of respondents	[%]
Rank		
One-star	15	11.5
Two-stars	24	18.3
Three-stars	51	39.0
Four-stars	40	30.5
Five-stars	1	0.8
The segment before the pandemic		
Primarily foreign clientele	17	13.0
Primarily Czech clientele	62	47.3
Only foreign clientele	1	0.8
Only Czech clientele	5	3.8
Approximately 50/50 foreign and Czech clientele	40	30.5
Primarily business clientele	14	10.7
Primarily leisure clientele	18	13.7
Only business clientele	2	1.5
Only leisure clientele	6	4.6
Approximately 50/50 business and leisure clientele	16	12.2
Other	2	1.5
Category		
Hotel	53	40.5
Hotel garni	4	3.1
Guesthouse	60	45,8
Spa hotel	2	1.5
Wellness hotel	8	6.1
Golf hotel	4	3.1
Locality		
Urban facility	50	38.2
Rural facility	53	40.5
Close to an airport	2	1.5
Close to an exhibition ground or conference center	3	2.3
Resort	16	12.2
Mountain	7	5.3

Source: Own questionnaire

The survey’s main objective was to show the changes in hotel pricing and sales strategies, hotel segments, and changes in product portfolio (e.g., the introduction of new packages). In this context, we also examined the impact on the company’s operations, especially the number of employees and the cost. We also examined how customer booking times had changed from the pre-pandemic period. Furthermore, we examined safety requirements (e.g., maintaining a safe distance, room service, or check-in/out apps), which were safety regulations approved by the Czech Ministry of Health at the time of data collection. The list of all used variables that were collected can be found below:

- Segment BEFORE the Pandemic,
- Segment DURING the Pandemic,
- Reservations BEFORE the Pandemic,
- Reservations DURING the Pandemic,
- Change in Pricing Strategy,
- Change in Sales Strategy,
- Reduction in Headcount,
- Cost Cutting,
- Hygiene Measures,
- Accommodation Packages.

### Data analysis

A dependency analysis was performed using Pearson’s chi-square test to examine the impact of the change in strategy on the individual characteristics and type of service establishments, including category, quality, location, and primary target segment before the pandemic (see Table 1). The dependency was tested at the 5% and 10% significance levels. Classification into quality levels was based on the establishment’s number of stars. The main criteria within the monitored pre-pandemic customer segment were nationality (domestic or foreign) and purpose of travel (business or leisure). The facilities’ tourism use (urban or rural) was monitored within the locality.

## Results

### General development of the Czech tourism

**Figure 1.**
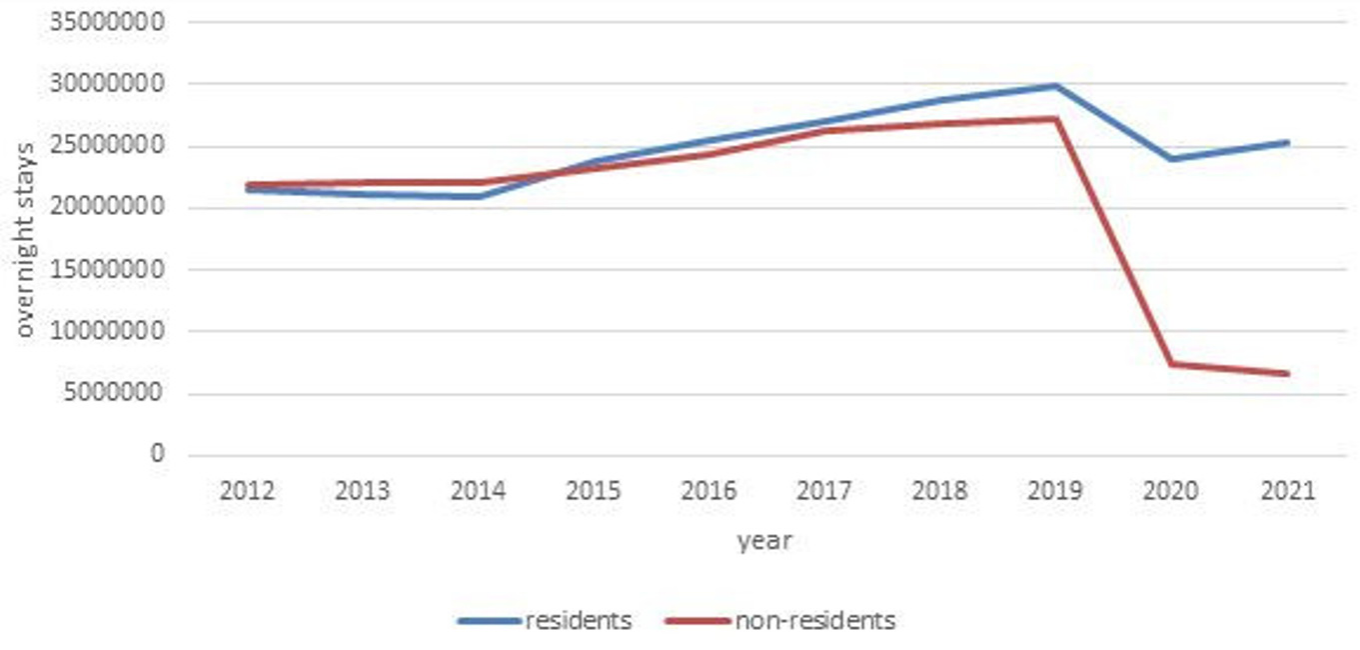
Number of overnight stays by tourists in Czechia from 2012 to 2021. Source: Public database of Czech Statistical Office Praha. Own elaboration.

Figure 1 shows the development of Czech tourism over the last 10 years. The slight increase in the number of overnight stays – with domestic and foreign tourists occupying approximately the same place in the market – was significantly interrupted by the onset of the COVID-19 pandemic. In 2020, there was a decrease in overnight stays by domestic and foreign tourists. In 2021, the number of overnight stays by domestic tourists began to increase, while the number of overnight stays by foreigners continued to decline. Additionally, the most common tourist accommodation in the Czech countryside is cottages, whose performance is not statistically monitored if they are not used commercially.

These results suggest that destinations aimed primarily at foreign tourists before 2020 were likely to have seen a significantly larger drop in tourism than destinations working mainly with domestic tourists. We examined this using the South-Moravian region as an example. We compared developments in its capital, Brno, with rural regions, represented by administrative districts of municipalities with extended power, except for the Brno-countryside district and areas of remaining district towns. We aimed to compare Brno with rural micro-regions. There was no municipality with more than 11,000 inhabitants in the defined area, and the comparison period was from 2019 to 2021.

### Differences between rural and urban areas

**Figure 2.**
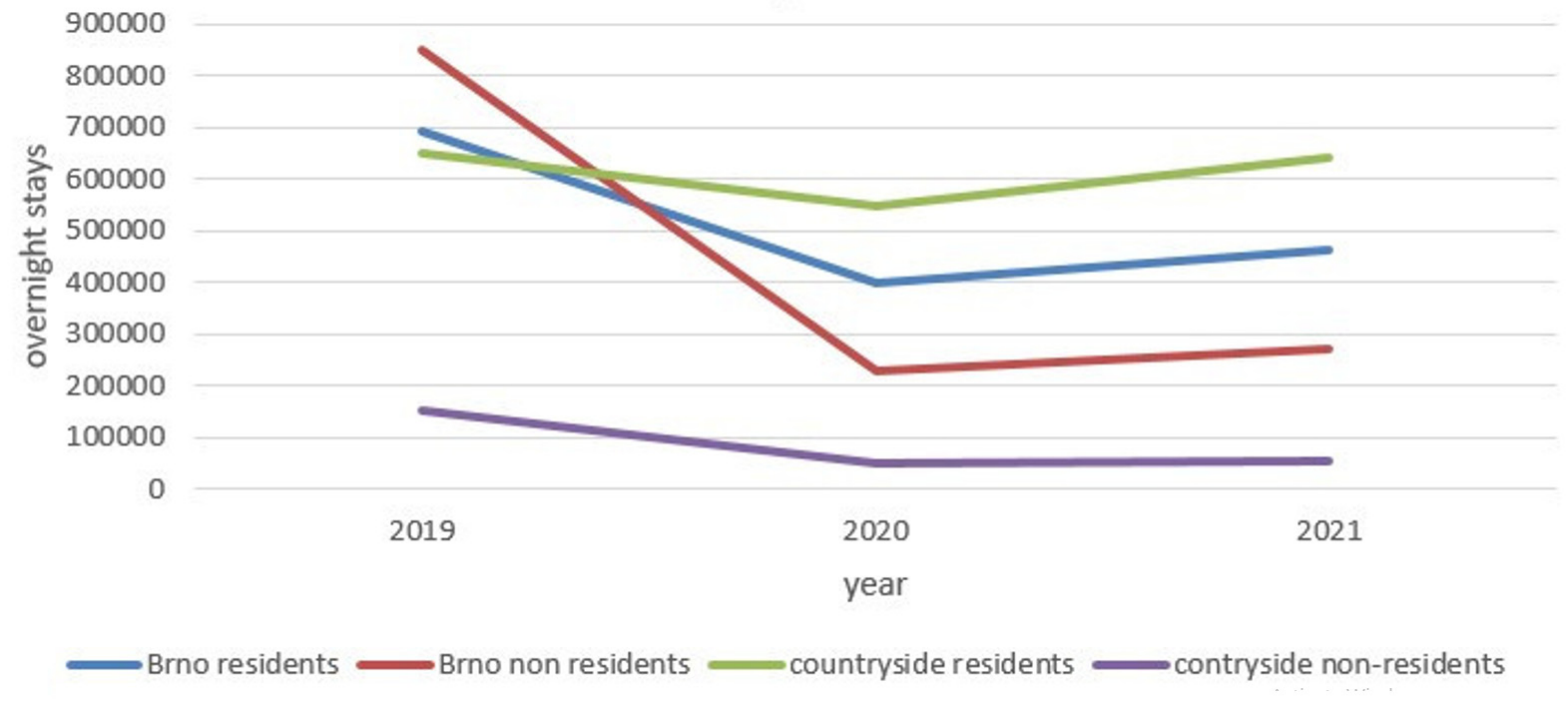
Overnight stays in selected destinations in the South-Moravian region 2019–2021. Source: Public database, Czech Statistical Office Prague. Own elaboration.

Figure 2 shows that in the final pre-COVID year, 2019, foreign visitors to Brno were the main tourist segment. The following year, their number dropped to a quarter of 2019 levels. It can be assumed that the main reason for this decline was the fall in conference tourism (Brno has six public and state universities) and commercial tourism (Brno is the largest trade fair center in Czechia). Domestic tourists became the strongest market segment in rural destinations.

The increase in domestic tourism was probably due to the limited opportunity to travel abroad. However, other reasons could exist, such as a greater sense of security or an uncertain financial outlook during the pandemic. In 2021, overnight stays slowly increased compared with 2020, while domestic guests in rural micro-regions were approaching pre-pandemic numbers. Currently, tourism in the South-Moravian region is based on domestic tourists, who visit mainly rural destinations. The number of foreign tourists remained low in 2021.

### Results of the questionnaire

The structure of the respondents according to their ranking and visitor segment is in Table 1. Over 72% of the surveyed hotels and guesthouses focused primarily on Czech clientele in 2021, 45.8% of respondents changed their pricing strategy, and 32.8% changed their sales strategy. A rapid increase in room bookings shortly before a trip was evident. Before the pandemic, only 8.4% of bookings were made shortly before travel, while 54.2% were made early during the pandemic. Accommodation packages were offered by 31.3% of facilities before the pandemic, 9.9% introduced them during the pandemic and 11.5% adapted them to the new situation. A total of 31% reduced their employee numbers, and 63.4% cut other costs. Capacity constraints and compliance dominated hygiene measures, followed by in-room services (22.1%), digital check-in and check-out (19.1%), and in-house contact control applications (8.4%). In 11.5% of cases, the company closed. Most respondents joined state aid schemes, but only 8.4% considered them sufficient.

Regarding pricing, there was a decrease in prices that could be explained by the change in clientele, especially the decrease in foreign and business customers, and an effort to replace these with domestic leisure customers. This was reflected in more special offers for domestic guests, especially in city hotels. Hotels began to offer a choice of dynamic rather than fixed rates to the business segment. The uncertain times were reflected in the offer of flexible cancellation terms for both the business and leisure segments.

In addition, a dependency analysis was performed to learn more about the strategy changes. Table 2 shows the relationships between these changes and the characteristics/type of service establishment. The dependency was tested at the 5% and 10% significance levels. ‘Yes+’ shows that the dependence of the given change on the establishment type or characteristic was proven at the 5% significance level, while ‘Yes’ shows that dependence was proven only at the 10% significance level. ‘No’ shows that the dependence was not proven even at the 10% level; thus, the strategy change did not depend on accommodation type or characteristics.

**Table 2.** Dependency analysis. Pearsons *χ* ^2^ test in the contingency table.

	Locality	Category of accommodation	Stars	Segment nationality (before)	Segment type (before)
**Price strategy**	Yes+	Yes+	Yes+	Yes	No
**Marketing strategy**	Yes+	Yes+	Yes+	x	x
**Change of costs**	x	No	x	x	x
**Decreasing number of** ** employee**	No	No	No	x	x
**Segment nationality (during)**	x	x	x	x	x
**Segment type (during)**	x	x	x	x	x
**Packages**	x	x	x	x	x
**Reservation (during)**	x	x	x	x	x

Source: own questionnaire

Table 2 and Table 3 show that changes in pricing strategy were related to an establishment’s location, category, and quality. Dependence on the hotel segment was only partial and was confirmed only for domestic and foreign tourists. Segmentation by travel purpose (leisure or business) was probably unimportant. Surprisingly, marketing strategy changes did not relate to segmentation. Headcount reduction was probably unrelated to facility type, location, and quality. Other dependencies were unfortunately not measurable due to the low number of responses in the category.

**Table 3.** P-values for changed in pricing strategy, marketing and segmentation.

P-values					
	Locality	Category of accommodation	Stars	Segment nationality (before)	Segment type (before)
**Price strategy**	0.0005	0.0000	0.0077	0.0503	0.8691
**Marketing strategy**	0.0029	0.0031	0.0093	0.0869	0.8007
**Change of costs**	0.5284	0.2993	0.1054	0.4585	0.3248
**Decreasing number of ** **employee**	0.8532	0.8843	0.2550	0.0052	0.6047
**Segment nationality (during)**	0.0337	0.1524	0.4020	0.0000	0.1543
**Segment type (during)**	0.2152	0.9825	0.7931	0.4051	0.1635
**Packages**	0.3193	0.0571	0.0010	0.1416	0.1495
**Reservation (during)**	0.0821	0.1385	0.1230	0.0261	X

Source: own questionnaire

## Discussion

Perceptions of travel risk and willingness to change travel plans increased significantly due to the COVID-19 pandemic ( Neuburger & Egger, 2021). According to Sánchez-Cañizares *et al.* (2021), this has negatively impacted tourists’ attitudes towards travel. It is important to build long-term trust with potential tourists. According to Shin and Kang (2020), trust in the hospitality industry could be improved by implementing technological innovations and risk mitigation strategies. Creating a safe and healthy environment for tourists and employees is also essential ( Stergiou & Farmaki, 2021; Yu *et al.,* 2018). Hence, per the Education during COVID-19 and beyond (2020) policy brief, hotels should provide a safe experience for their guests.

The study showed that the tourism industry suffered significant losses from anti-pandemic measures; however, these losses were not evenly distributed. The largest decrease in tourist numbers was recorded by destinations previously aimed at foreign tourists. These were mainly large cities, UNESCO-listed monuments, or spas. Conversely, rural destinations suffered less and were strengthened relatively, sometimes absolutely even on a national scale. Other researchers have arrived at a similar conclusion ( Chin, 2022; Cvijanović *et al.*, 2021; Kupi & Szemerédi, 2021; Peixeira Marques *et al.*, 2021).

According to data from the Czech Statistical Office, in the first half of 2022, the number of domestic tourists has already exceeded the last pre-COVID year of 2019, while the number of foreign visitors has not yet reached the pre-COVID level. The largest number of non-residents came from Germany (755 thousand). They are followed by Slovaks (327 thousand), Poles, citizens of the USA and Great Britain, Austria, and Ukraine ^2^. The largest numbers of foreign visitors come from neighbouring countries. Russians and tourists from Asia are almost completely absent, although they were not numerically significant, but they spent the most per capita.

Although this conclusion seems logical, the question is whether and to what extent the trend towards relocation to rural regions could be at least partially maintained, which could relieve some congested areas ( Kotsila *et al.,* 2021), and lead tourism to become more ethical, responsible and sustainable ( Higgins-Desbiolles, 2020). Cheer (2020) sees the future of tourism as ‘human flourishing’. Alraouf (2021) speaks of a new normality. Arguments supporting the development of domestic tourism include higher security, cheaper stays, the absence of language problems, discovering one’s own country, and the possibility of traveling independently.

The data reported by the survey were the operational activities of individual entrepreneurs who responded to a rapid change in their situations. The survey findings reveal significant insights into the hotel sector, with implications extending beyond Czechia to similar regions. The correlation between changes in pricing strategies and factors like location, category, and quality suggests that hotels need to tailor their pricing based on their specific context. For instance, luxury hotels in prime locations may adopt different pricing tactics compared to budget hotels in non-central areas. This trend can guide hoteliers in other regions to consider their unique market positions when revising pricing strategies, ensuring they remain competitive and appealing to their target demographics.

The partial dependence of pricing strategies on hotel segments, specifically for domestic versus foreign tourists, indicates a nuanced approach to pricing. This suggests that while segmentation is somewhat relevant, it may not be the primary driver for pricing decisions. Hotels in other regions might similarly find that focusing on broader categories like domestic and international guests is more practical than segmenting by travel purposes.

The findings that headcount reduction appears unrelated to the type, location, and quality of facilities suggests that staff adjustments might be driven more by overarching economic conditions or internal financial strategies rather than specific hotel characteristics. For the broader sector, this indicates that workforce management strategies during crises might need a more standardized approach, focusing on financial sustainability rather than tailoring to specific hotel attributes.

The difficulty in measuring other dependencies due to low response rates highlights a potential challenge in gathering comprehensive data. This underscores the importance of robust data collection methods and higher participation rates in surveys for more conclusive results. For similar regions, it suggests the significant need for industry collaboration and data sharing to better understand and respond to sector-wide changes.

Strategic measures at the destination level would have to be taken to maintain the trend toward shifting from congested to rural regions. As Milano and Koens (2022) stated, to come to more balanced tourism, it is necessary to not only come up with alternative visions and strategies-, but also to engage with the political economy nature of tourism development. Since these regions are generally not very popular and generally less equipped with infrastructure, advertising, branding and image building are crucial. For this, digital possibilities must definitely be used.

Unfortunately, we must also focus on another disruptive factor for international tourism ( Pandey & Kumar, 2022): the consequences of Russia’s aggression against Ukraine. This may manifest primarily by closing borders for citizens of some countries and filling accommodations with refugees, and secondarily by causing economic problems for Czech households, limiting their opportunities, especially hosting foreign tourists.

In addition, the energy crisis is beginning to manifest itself, which will undoubtedly make services more expensive. In 2020, Chang, McAleer and Ramos wrote that tourism, together with the energy industry, is one of the world's largest employers, but unlike energy, it suffers from the risk of shock developments. Two years later, it turns out that the energy sector is also going through a shocking development, which undoubtedly has a retroactive effect on the tourism industry as well.

Data on the number of overnight stays are official data from the Czech Statistical Office. Their relevance is reviewed according to the criteria of the statistical service. A limiting factor is the fact that these data only affect mass accommodation facilities and therefore do not cover the entire segment of the tourism industry. Data from the questionnaire survey recorded a return of around 20%. However, due to the small willingness of the business sector to engage in questionnaire surveys, the return is relatively high. It can be judged that among the respondents there were rather providers who are interested in the development of the branch and also larger entrepreneurs who have an administrative apparatus that would generate answers. Due to the method of data collection, there was a lack of a face-to-face element that could reveal the possible falsity of the answers. In general, individuals tend to present themselves better than they really are, while firms might tend to highlight problems with the subconscious goal of gaining external support.

## Conclusion

The increasing importance of domestic tourism and the reduction in destinations aimed at foreign tourists were confirmed. Some operational measures of accommodation facilities responding to the situation were noted, predominantly in the price and marketing spheres. Survey findings suggested that factors like location and quality play a crucial role in shaping these strategies. This can help hotels in Czechia and similar regions adopt more informed and effective strategies, particularly in pricing, marketing, and workforce management, ensuring they are better prepared for future challenges. For further development, it would be appropriate to move from operational to strategic measures, which a vision should precede. These strategies should be linked to destination management rather than individual business measures. According to the sociological survey (organized by FOCUS Agency), holidays through tourism are common for more than 80% of Czech households. About a third spent it domestically in 2021, more than a fifth abroad, and 30% of households combined both forms.

One of the trends that emerged in tourism in the context of anti-covid measures was an emphasis on orientation towards host communities ( Kamata, 2022; Lapointe, 2020). Several authors deal with visions that assume that going back is no longer possible and that tourism will develop differently in the post-COVID era, although there are different ideas about what this difference will consist of ( Lew *et al.,* 2020). Some authors ( Higgins-Desbiolles *et al.*, 2022) are inclined to the opinion that the trend will be from corporate to socializing tourism. Some authors ( Crossley, 2020) believe in the greening of tourism, consisting, among other things, of relief for congested destinations.

Like any crisis, the current one is also an opportunity to reassess existing procedures and accelerate the introduction of innovations. Both tourism providers and tourists will look for ways to adapt to the new conditions. Some providers may not make it, but others will take their place. The development of tourism belongs to the basic trends of the transition to a post-productive society.

During further research, it is advisable to take into account that doing business in the tourism industry is relatively risky. The return on investments depends not only on the weather and fashion trends but also on the development of the global and local situation. Issues of war and peace, terrorism, uncontrolled migration, the energy crisis, spin-offs of the Green Deal may play a role similar to the COVID-19 pandemic. Overtourism can also come into play in some regions. A question for future research may be the resilience of urban destinations to rural ones.

We assume that domestic tourists who consider vacation as part of their standard will be willing to leave it. If they find themselves in a disadvantageous financial situation, they will probably try to replace expensive and complicated (foreign) destinations with cheaper and more accessible domestic destinations. Another issue is the return of foreign tourists. We can probably expect a strengthening of currents from the surrounding states, which can be reached by car. This will enhance the independence and creativity of tourists. The influx of tourists from more distant countries - especially overseas ones will probably decrease, and its return is a question of the overall development of the geopolitical situation and globalization tendencies.

When and to what level the tourist industry stabilizes is not yet entirely clear. However, these changes represent an opportunity for tourism to become more sustainable. Any changes in this direction would be accompanied by economic issues (foreign tourists usually spend more money), infrastructure investment, and organizational and transport changes. Restrictions on air transport (if maintained) are certainly positive from an environmental perspective. However, the increased emphasis on individual car transport may have negative consequences. Similarly, there is a positive reduction in pressure on areas congested with tourists, but diverting them to rural areas may cause other difficulties. Therefore, tourism development and related externalities would be a worthwhile research subject in the future. For example, Assaf, Kock and Tsionas (2022) set out an agenda for future research.

## Data Availability

***Underlying data*** ZENODO: Milada Šťastná, Kateřina Ryglová, Antonín Vaishar, Andrea Králíková. (2023). HORIZONT SPOT; dataset The impact of anti-COVID measures on accommodation performance (Open Research Europe) [Data set]. Zenodo. https://doi.org/10.5281/zenodo.10361377 [ Šťastná *et al.*, 2023]. The project contains the following underlying data: Survey data [survey_data_JMK.xlsx] (Raw Czech data from questionnaire survey in excel). English version ***Extended data*** ZENODO: Milada Šťastná, Kateřina Ryglová, Antonín Vaishar, Andrea Králíková. (2023). HORIZONT SPOT; dataset The impact of anti-COVID measures on accommodation performance (Open Research Europe) [Data set]. Zenodo. https://doi.org/10.5281/zenodo.10361377 [ Šťastná *et al.*, 2023]. The project contains the following extended data: Dotaznik.pdf [questionnaire in Czech]. TheQuestionaire.pdf [questionnaire translated into English] Data are available under the terms of the Creative Commons Attribution 4.0 International license (CC-BY 4.0).
