# Supplementary material for: The impact of anti-COVID measures on accommodation performance
Source: Open Res Eur. 2024 Jul 29;4:40. Originally published 2024 Feb 28. [Version 2] doi: 10.12688/openreseurope.16566.2 (PMC11325133; doi:10.12688/openreseurope.16566.2)
Supplement: Peer review history

# Reviewer report: Reviewer response for version 2

**DOI:** 10.21956/openreseurope.19699.r42560

**Published:** 2024-08-14

**Authors:** Maria de Lurdes Calisto

**Affiliations:** 1 Centre for Tourism Research, Development, and Innovation (CiTUR), Centre for Advanced Studies in Management and Economics (CEFAGE), Estoril Higher Institute for Tourism and Hotel Studies, Estoril, Portugal

I consider the revision made to the article sufficient for its approval.

If applicable, is the statistical analysis and its interpretation appropriate?

Partly

Is the study design appropriate and is the work technically sound?

Yes

Is the work clearly and accurately presented and does it engage with the current literature?

Yes

Are the conclusions drawn adequately supported by the results?

Partly

Are sufficient details of methods and analysis provided to allow replication by others?

Partly

Are all the source data and materials underlying the results available?

Yes

Reviewer Expertise:

Tourism & Hospitality Business Management, with a focus on Strategy, Marketing, Innovation and Entrepreneurship.

I confirm that I have read this submission and believe that I have an appropriate level of expertise to confirm that it is of an acceptable scientific standard.

# Reviewer report: Reviewer response for version 2

**DOI:** 10.21956/openreseurope.19699.r42559

**Published:** 2024-07-30

**Authors:** Lóránt Dénes Dávid

**Affiliations:** 1 Faculty of Economics and Business, John von Neumann University, Kecskemet, Hungary; 2 Institute of Rural Development and Sustainable Economy, Hungarian University of Agriculture and Life Sciences, Godollo, Hungary

I accept the article.

If applicable, is the statistical analysis and its interpretation appropriate?

Yes

Is the study design appropriate and is the work technically sound?

Yes

Is the work clearly and accurately presented and does it engage with the current literature?

Yes

Are the conclusions drawn adequately supported by the results?

Yes

Are sufficient details of methods and analysis provided to allow replication by others?

Yes

Are all the source data and materials underlying the results available?

Yes

Reviewer Expertise:

tourism, hospitality, geography, earth sciences, economics, environmentalism, regional studies and sciences, sustainable development, circlular economy, bioeconomy, multi-, trans- and interdisciplinary fields

I confirm that I have read this submission and believe that I have an appropriate level of expertise to confirm that it is of an acceptable scientific standard.

# Reviewer report: Reviewer response for version 1

**DOI:** 10.21956/openreseurope.17881.r40403

**Published:** 2024-06-07

**Authors:** Maria de Lurdes Calisto

**Affiliations:** 1 Centre for Tourism Research, Development, and Innovation (CiTUR), Centre for Advanced Studies in Management and Economics (CEFAGE), Estoril Higher Institute for Tourism and Hotel Studies, Estoril, Portugal

The study titled "Impact of the COVID-19 Pandemic on the Hotel Industry in Czechia" delves into how the pandemic has reshaped the hotel and tourism sectors in this Central European nation. The research was conducted under ethical guidelines approved by Mendel University in Brno and involved a comprehensive questionnaire survey sent to lodging establishments across Czechia.

 The study highlights that the pandemic forced unprecedented adaptability from tourism service providers. With travel restrictions and health measures in place, domestic tourism gained relevance compared to international tourism, which suffered significantly.

 Data for the study were collected through a questionnaire sent to 666 lodging establishments in March 2021, achieving a 20% response rate. The survey focused on several aspects, including changes in consumer behaviour, occupancy rates, cost reductions, employee numbers, sales and pricing strategies, and shifts in target clientele. Secondary data on overnight stays from 2012 to 2021 were sourced from the Czech Statistical Office.

 The study underscores the pandemic's uneven impact on tourism, with rural areas proving more resilient due to their domestic focus. It highlights the need to build long-term trust with tourists through technological innovations and safety measures. The findings suggest that while domestic tourism has growth potential, strategic measures are necessary to sustain this trend and mitigate future disruptions.

 In summary, this research provides valuable insights into the adaptive strategies of the Czech hotel industry during the COVID-19 pandemic. It emphasizes the importance of flexibility and innovation in overcoming challenges and suggests that fostering domestic tourism could lead to a more balanced and sustainable future for the sector. The study's limitations include the partial representation of the tourism sector, given its focus on mass accommodation facilities, and the inherent biases in self-reported data.

 The article could also benefit from some improvements:

- The introduction should more clearly outline the study's objectives and what the reader can expect to learn.

- Provide more detailed information about the survey design.

- Provide more context for the statistical tests used, and explain what the results mean in practical terms.
- Discuss the broader implications of the findings for the sector, both in Czechia and potentially in other similar regions.
- Strengthen the conclusion by summarizing the essential findings and their significance, providing clear, actionable recommendations for tourism operators and suggesting areas for future research.

Nonetheless, this study offers a comprehensive overview of the pandemic's impact and potential strategies for recovery and resilience in the tourism industry and is relevant for the academic and professional communities.

If applicable, is the statistical analysis and its interpretation appropriate?

Partly

Is the study design appropriate and is the work technically sound?

Yes

Is the work clearly and accurately presented and does it engage with the current literature?

Yes

Are the conclusions drawn adequately supported by the results?

Partly

Are sufficient details of methods and analysis provided to allow replication by others?

Partly

Are all the source data and materials underlying the results available?

Yes

Reviewer Expertise:

Tourism & Hospitality Business Management, with a focus on Strategy, Marketing, Innovation and Entrepreneurship.

I confirm that I have read this submission and believe that I have an appropriate level of expertise to confirm that it is of an acceptable scientific standard, however I have significant reservations, as outlined above.

# Reviewer report: Reviewer response for version 1

**DOI:** 10.21956/openreseurope.17881.r40400

**Published:** 2024-05-17

**Authors:** Lóránt Dénes Dávid

**Affiliations:** 1 Faculty of Economics and Business, John von Neumann University, Kecskemet, Hungary; 2 Institute of Rural Development and Sustainable Economy, Hungarian University of Agriculture and Life Sciences, Godollo, Hungary

The article titled "The Impact of Anti-COVID Measures on Accommodation Performance" provides valuable insights into the effects of the COVID-19 pandemic on tourism in Czechia, specifically focusing on mass accommodation facilities. 

 In the wake of the unprecedented challenges posed by the COVID-19 pandemic, "The Impact of Anti-COVID Measures on Accommodation Performance" emerges as a beacon of understanding, shedding light on the intricate dynamics of Czech tourism during this tumultuous period.

 One of the most commendable aspects of this article is its meticulous methodology, which employed a questionnaire survey and rigorous statistical analysis to gather data from 131 hotels and guesthouses across Czechia. This comprehensive approach not only ensures the reliability of the findings but also underscores the scholarly rigor of the research.

 The results of the study offer invaluable insights into the shifting landscape of tourism in the face of the pandemic. Particularly noteworthy is the confirmation of the increasing significance of domestic tourism and the heightened vulnerability of destinations reliant on foreign visitors. By highlighting these trends, the article provides a nuanced understanding of the challenges and opportunities facing the tourism sector in Czechia.

 Moreover, the article's emphasis on the need for strategic, destination-focused approaches to tourism management is both timely and insightful. By advocating for a shift from operational to strategic measures, the authors offer a forward-thinking roadmap for revitalizing the tourism industry in the post-pandemic era. Furthermore, the call for greater emphasis on environmentally friendly and sustainable tourism practices underscores the article's commitment to fostering a resilient and responsible tourism sector.

 In conclusion, "The Impact of Anti-COVID Measures on Accommodation Performance" stands as a valuable contribution to the scholarly discourse on tourism management in times of crisis. Its rigorous methodology, insightful findings, and forward-looking recommendations make it essential reading for academics, policymakers, and industry practitioners alike.

 More citations are needed.

If applicable, is the statistical analysis and its interpretation appropriate?

Yes

Is the study design appropriate and is the work technically sound?

Yes

Is the work clearly and accurately presented and does it engage with the current literature?

Yes

Are the conclusions drawn adequately supported by the results?

Yes

Are sufficient details of methods and analysis provided to allow replication by others?

Yes

Are all the source data and materials underlying the results available?

Yes

Reviewer Expertise:

tourism, hospitality, geography, earth sciences, economics, environmentalism, regional studies and sciences, sustainable development, circlular economy, bioeconomy, multi-, trans- and interdisciplinary fields

I confirm that I have read this submission and believe that I have an appropriate level of expertise to confirm that it is of an acceptable scientific standard.

## References

[1] : OPPORTUNITIES FOR THE DEVELOPMENT OF INNOVATION AMONG HOTELS IN NORTHERN HUNGARY. *GeoJournal of Tourism and Geosites* .2022;40(1) : 10.30892/gtg.40132-828 267-273 10.30892/gtg.40132-828

[2] : Google and Apple mobility data as predictors for European tourism during the COVID-19 pandemic: A neural network approach. *Equilibrium. Quarterly Journal of Economics and Economic Policy* .2023;18(2) : 10.24136/eq.2023.013 419-459 10.24136/eq.2023.013

[3] : The Positive Effect of Workplace Accommodation on Creative Performance of Employees With and Without Disabilities. *Front Psychol* .2020;11: 10.3389/fpsyg.2020.01217 1217 10.3389/fpsyg.2020.01217 32625141 PMC7311633

[4] : Accommodations and academic performance: First-year university students with disabilities. *Canadian Journal of Higher Education* .2021;51(1) : 10.47678/cjhe.vi0.188985 41-56 10.47678/cjhe.vi0.188985
